# Detection of hepatitis E virus in wild boars of rural and urban regions in Germany and whole genome characterization of an endemic strain

**DOI:** 10.1186/1743-422X-6-58

**Published:** 2009-05-14

**Authors:** Anika Schielke, Katja Sachs, Michael Lierz, Bernd Appel, Andreas Jansen, Reimar Johne

**Affiliations:** 1 Federal Institute for Risk Assessment, Diedersdorfer Weg 1, 12277 Berlin, Germany; 2 State Office for Food Safety and Consumer Protection Thuringia, Bad Langensalza, Germany; 3 Free University of Berlin, Faculty for Veterinary Medicine, Germany; 4 Robert Koch Institute, Department for Infectious Disease Epidemiology, Berlin, Germany

## Abstract

**Background:**

Hepatitis E is an increasingly diagnosed human disease in Central Europe. Besides domestic pigs, in which hepatitis E virus (HEV) infection is highly prevalent, wild boars have been identified as a possible source of human infection. In order to assess the distribution of HEV in the wild boar population of Germany, we tested liver samples originating from different geographical regions for the presence of the HEV genome and compared the detected sequences to animal and human HEV strains.

**Results:**

A total of 148 wild boar liver samples were tested using real-time RT-PCR resulting in an average HEV detection rate of 14.9% (95% CI 9.6–21.6). HEV was detected in all age classes and all geographical regions. However, the prevalence of HEV infection was significantly higher in rural as compared to urban regions (p < 0.001). Sequencing of the PCR products indicated a high degree of heterogenicity of the detected viruses within genotype 3 and a grouping according to their geographical origin. The whole genome sequence of an HEV isolate (wbGER27) detected in many wild boars in the federal state of Brandenburg was determined. It belongs to genotype 3i and shows 97.9% nucleotide sequence identity to a partial sequence derived from a human hepatitis E patient from Germany.

**Conclusion:**

The results indicate that wild boars have to be considered as a reservoir for HEV in Germany and that a risk of HEV transmission to humans is present in rural as well as urban regions.

## Background

Hepatitis E virus (HEV) causes a human disease with acute hepatitis as the major clinical symptom. Although the case-fatality rate of hepatitis E is low in the general population, rates up to 25% have been observed in pregnant women [1]. In developing countries, HEV infection is one of the most important causes of infectious hepatitis leading to epidemics associated with contaminated water resources [2]. The hepatitis E cases in North America and Central Europe could be either traced to imported infections from endemic regions or to autochthonous HEV infections [3-5]. In Germany, an increasing number of non-travel related hepatitis E cases have been notified in the last years leading to an increase from 44% of 54 hepatitis E cases in 2005 to 63% of 73 hepatitis E cases in 2007 for the autochthonous infections [6].

HEV is a single-stranded RNA virus and the only member of the unassigned genus *Hepevirus *[7]. Until now, four genotypes and several subtypes have been defined [8]. Genotypes 1, 2 and 4 are found only in distinct geographical regions of the world whereas genotype 3 seems to have a worldwide distribution [8]. Among genotype 3 and 4, HEV strains closely related to human HEV have been detected in pigs, deer and wild boar indicating the possibility of a zoonotic transmission [2,9,10]. HEV strains isolated from pigs in the Netherlands have been shown to be closely related to HEV strains from human cases of hepatitis E of the same region indicating that autochthonous HEV infections may be acquired from pigs in Central Europe [4,11,12].

Wild boars (*Sus scrofa*) have shown a significant increase in the population density throughout Europe and the USA over the past decades. Subsequently, migration to urban areas and close contact between wild boars and humans has been observed [13]. In Berlin, the capital city of Germany, the estimated number of wild boars living in urban areas is 5.000 animals [13]. Reports on human hepatitis E cases after consumption of uncooked meat from wild boar strengthened the hypothesis of a zoonotic origin of human HEV infections [14-16]. In Japan, wild boars have been suggested to serve as a reservoir for HEV infections as a broad variety of strains including those closely related to human HEV strains has been detected in this animal species [9]. A high prevalence of HEV infection was demonstrated in a wild boar population of Italy [17]. In Germany, HEV sequences have been detected in archived sera of wild boar originally sampled in 1995/1996 demonstrating that the virus has been present in the wild animal population for a longer time [18,19]. Recently, consumption of wild boar meat has been identified as a risk factor for autochthonous HEV infections in Germany [6].

In order to determine the actual distribution of HEV in wild boars from Germany, liver samples were tested for the presence of HEV and subsequently genotyped. By comparing samples derived from different urban and rural regions, possible differences in the epidemiology of the infections were investigated. The availability of the generated HEV sequences may serve as a basis for comparing actual and future human isolates to identify transmission events between wild boar and humans.

## Methods

### Samples

Liver tissue samples were collected from wild boars hunted in the study area (federal states of Brandenburg and Thuringia, cities of Berlin/Potsdam) for population control between 2005 and 2008. Wild boars were categorized according to age (teeth method; shoats: <1 year, yearlings: 1–2 years, adults: >2 years), sex, and location of death for most samples. Wild boar samples were considered to originate from urban areas in case that they have been sampled in settled areas (as defined by administrative districts) of more than 10,000 people. The remainder samples were considered to originate from rural areas. All samples had been stored at -80°C until analysis.

### RNA extraction and PCR analysis of samples

RNA was isolated from liver suspensions using the RNeasy Mini Kit (Qiagen, Hilden, Germany) along with QIAshredder collumns (Qiagen, Hilden, Germany) according to the manufacturer's protocol. The extracted RNA was tested by real-time RT-PCR according to Jothikumar et al. [20] in an ABI PRISM 7500 cycler using the Quantitect Probe RT-PCR Kit (Qiagen, Hilden, Germany). Positive samples were additionally tested by RT-PCR according to Schlauder et al. [21] and modified by Herremans et al. [4] amplifying a 197 bp product of open reading frame (ORF)-2 using the One-Step RT-PCR Kit (Qiagen, Hilden, Germany). For amplification of a 287 bp product of ORF-1, a nested RT-PCR was performed according to Preiss et al. [5] using the One-Step RT-PCR Kit (Qiagen, Hilden, Germany) for the first round of RT-PCR and the TaKaRa Ex Taq (Takara Bio Europe S.A.S., Saint-Germain-en-Laye, France) for the nested PCR. PCR products were separated on ethidium bromide-stained 1.5% agarose gels and visualized by UV light.

### Amplification of the whole genome sequence of isolate wbGER27

The genome of isolate wbGER27 was amplified by RT-PCR in seven parts and by application of RACE protocols. First, four PCR-products were generated using the primer sets 1, 3 and 5 previously described by Xia et al. [22]. Then, primers ORF2F (5'-ACG TCT AGA ATG TGC CCT AGG GCT KTT CTG-3', nt 5172–5192, nucleotide numbering according to wbGER27) and ORF2R (5'-ACG TCT AGA TTA AGA CTC CCG GGT TTT RCC YAA-3', nt 7154–7131) were used to amplify the complete ORF-2-encoding region (constructed on the basis of an alignment of 24 HEV full-length sequences, not shown). Based on the sequences determined for these PCR products, specific primer pairs were constructed (5'-CCC GGT CGA CAG AGG TGT ATG T-3' [nt 870–890] and 5'-CAT CAA AAA CAA GCA CCC TTG GG-3' [nt 1382–1360]; 5'-ATT CAT GCA GTG GCT CCT GAT T-3' [nt 2606–2627] and 5'-ATC ACG AAA TTC ATA GCA GTG TG-3' [nt 4681–4659]) for amplification of the remaining parts of the genome. For RACE amplification of the 3'-end of the wbGER27 genome, reverse transcription was performed using the primer pA1 (5'-CCG AAT TCC CGG GAT CCT_17 _V-3', complementary to poly A tail), followed by PCR with primers 5'-CCG AAT TCC CGG GAT CC-3' (binding site on primer pA1) and 5'-ATT CGG CTC TTG CAG TCC TTG A-3' (nt 6982–7003). For RACE amplification of the 5'-end of the wbGER27 genome, the 5' RACE System Kit (Invitrogen GmbH, Karlsruhe, Germany) was used according to the supplier protocol with the gene-specific primers 5'-CCA ACT GCC GGG GTT GCA TCA A-3' (nt 191–170) and 5'-GAA TCT CAG TTT GCA CAC GAG A-3' (nt 161–140). All RT-PCRs were performed using the QIAGEN LongRange 2Step RT-PCR Kit (Qiagen, Hilden, Germany). Reverse transcription was carried out in a 20 μl reaction at 42°C for 90 min. PCR was subsequently performed in a 2720 Thermal Cycler (Applied Biosystems, Foster City, USA) using 5 μl of cDNA in 50 μl reactions and 93°C for 3 min, 35 cycles of 93°C for 30 sec, 56°C for 30 sec and 68°C for 5 min, and a final incubation at 68°C for 7 min.

### Sequence analysis

RT-PCR products considered for sequence analysis were purified using the Qiaquick DNA purification kit (Qiagen, Hilden, Germany) and subsequently cloned into the vector pCR4-TOPO using the TOPO TA Cloning Kit for Sequencing (Invitrogen GmbH, Karlsruhe, Germany). The inserts of the plasmids were sequenced using M13 Forward and M13 Reverse primers (Invitrogen GmbH, Karlsruhe, Germany) as well as gene-specific primers in an ABI 3730 DNA Analyzer (Applied Biosystems, Foster City, USA). The sequence of the wbGER27 genome was assembled from the determined sequence pieces using the SeqBuilder module of the DNASTAR software package (Lasergene, Madison, USA) and submitted to the GenBank database with accession number FJ705359. The partial sequences determined here were deposited with GenBank accession numbers FJ748515 – FJ748531. Sequence similarity searches were performed using the BLAST 2.2.14 search facility [23] and the GenBank database. Phylogenetic trees were constructed on the basis of the nucleotide sequences using the MegAlign module of the DNASTAR software package (Lasergene, Madison, USA) with the CLUSTAL W method and a bootstrap analysis with 1000 trials and 111 random seeds.

### Statistical analysis

For comparison of categorical variables between groups, we used the summary χ^2 ^test and Fisher's exact test. Calculations were done using Intercooled Stata 10 software (Stata Corporation, Texas, USA). A p-value of <0.05 was considered significant. The exact binomial method was used to calculate 95% confidence intervals of single proportions.

## Results

### Detection of HEV RNA in wild boar liver samples from Germany

**Figure 1:**
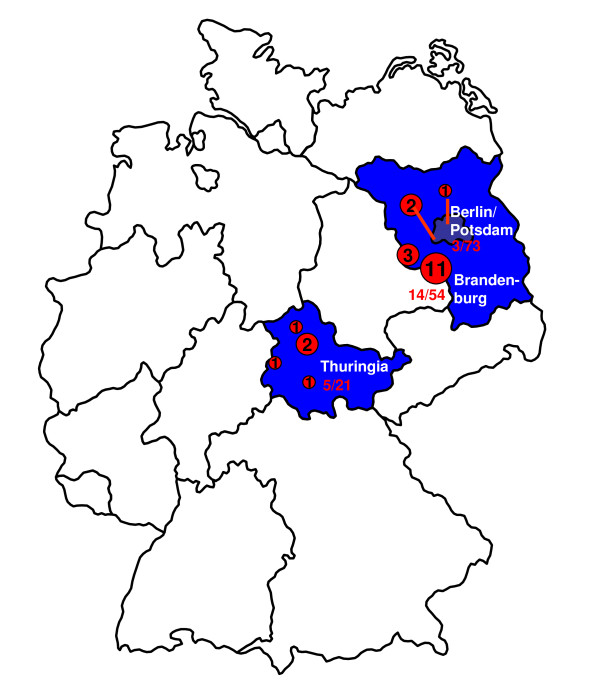
**Geographical origin of wild boar samples**. In the map of Germany, the federal states of Brandenburg and Thuringia are coloured in blue, the cities of Berlin/Potsdam are in dark blue. The areas, in which HEV positive wild boars have been detected, are marked by red circles containing the number of positive animals. The total number of positives out of all samples investigated from a federal state or from the cities is indicated by red numbers.

A total of 148 liver samples from wild boar originating from different regions of Germany were analysed by real-time RT-PCR for the detection of the HEV genome. By this, 22 samples were tested positive resulting in an overall detection rate of 14.9% (95%CI 9.6–21.6). A detailed analysis showed that 14 out of 54 (25.9%; 95%CI: 14.9–39.7) and 5 out of 21 (23.8%; 95%CI 8.2–47.2) were tested positive in the rural areas of the federal states of Brandenburg and Thuringia, respectively. In the cities of Berlin/Potsdam, 3 out of 73 (4.1%; 95%CI 0.9–11.6) wild boars were tested positive. The difference of detection rates among wild boars originating from rural vs. urban areas was highly significant (p < 0.001). The distinct distribution of positively and negatively tested areas is shown in Figure 1. The detection rate was highest in shoats (19,7%) and adult animals (12,9%), while 5,9% of yearlings were tested positive for HEV. The detection rate was unrelated to sex (p = 0.1).

### Genotyping of detected HEV strains

**Figure 2:**
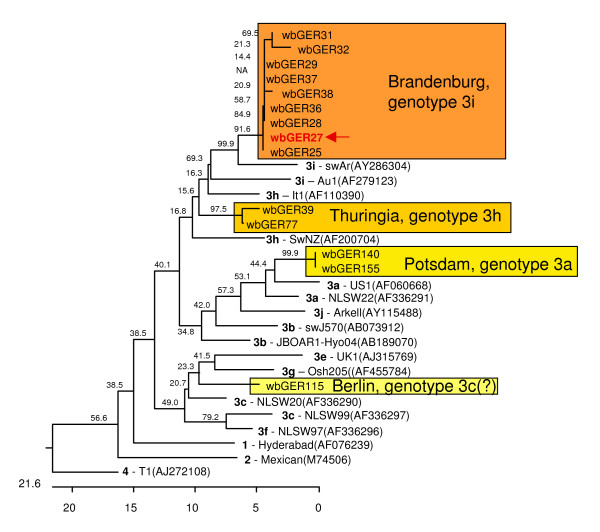
**Genotyping of wild boar HEV strains**. The phylogenetic tree was constructed based on a 148 base pair nucleotide sequence of ORF-2 using reference sequences. The genotypes according to Lu et al. [8] are indicated in bold face. The actual isolates from wild boars are marked in coloured boxes with respect to their geographical origin and deduced genotype. Isolate wbGER27, which was selected for whole genome sequencing, is shown in red and marked by a red arrow. The tree is scaled in nucleotide substitution units.

The positive samples were further analysed by RT-PCR amplifying a 197 bp fragment of ORF-2. Bands of the expected length could be detected in 14 out of the 22 samples and the DNA sequence could be determined. Phylogenetic analysis of the 148 bp sequence (excluding the primer sequences) indicated that all isolates belonged to genotype 3. Further subtyping was performed by comparison with prototype sequences of genotype 3 subtypes [8]. Although the resulting phylogenetic tree (Figure 2) generally shows low bootstrap values, which is most probably due to the short sequence used, a grouping according to the assigned subtypes is evident for the prototype strains. The sequences of wild boars clustered within different subgroups according to their geographical origin: the 9 sequences from Brandenburg clustered in genotype 3i, the two sequences from Thuringia clustered in genotype 3h, the two sequences from Potsdam clustered in genotype 3a and the isolate from Berlin branched between genotypes 3c and 3g.

### Comparison of HEV sequences to human HEV strains from Germany

**Figure 3:**
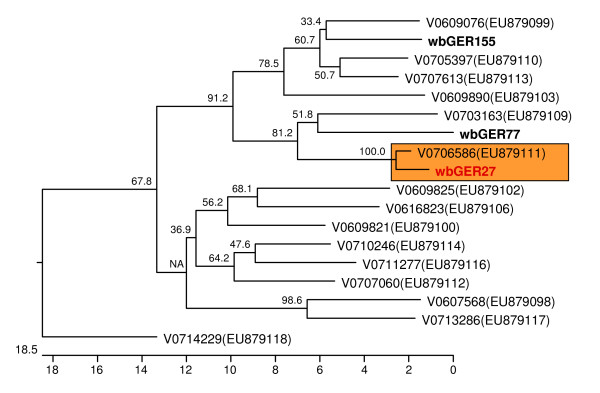
**Phylogenetic relationship between genotype 3 HEV strains derived from wild boars and humans from Germany**. The tree was constructed on the basis of a 287 bp sequence fragment of ORF-1. The actual isolates from wild boars are shown in bold face. The closely related isolates wbGER27 from wild boar and V0706586 from human are indicated with a coloured box. The tree is scaled in nucleotide substitution units.

To enable a comparison of the wild boar isolates with human HEV isolates derived from autochthonous infections acquired in Germany, sequences were retrieved from the GenBank database. As only partial sequences of ORF-1 were available, amplification of the corresponding region was tried by nested RT-PCR analysis of the positively tested wild boar samples. A PCR product with the expected length was only detected in three cases (isolates wbGER27, wbGER77 and wbGER155). As these samples also had shown the lowest ct values in real-time RT-PCR, the amount of HEV genome may be considered as the limiting factor for a positive ORF-1 PCR. The PCR products were compared to sequences of 15 genotype 3 isolates derived from recent human hepatitis E cases from Germany [6]. A very close relationship between the wild boar isolate wbGER27 and the human isolate V0706586 is evident from the phylogenetic tree (Figure 3), which reflects 97.9% nucleotide sequence identity between both strains within the 287 bp fragment analysed. With 92.1% nucleotide sequence identity, the human isolate V0609076 was most closely related to the wild boar isolate wbGER155. The human isolate V0703163 and the wild boar isolate wbGER77 showed 89.7% nucleotide sequence identity.

### Determination and sequence analysis of the full-length genome of wbGER27

**Figure 4:**
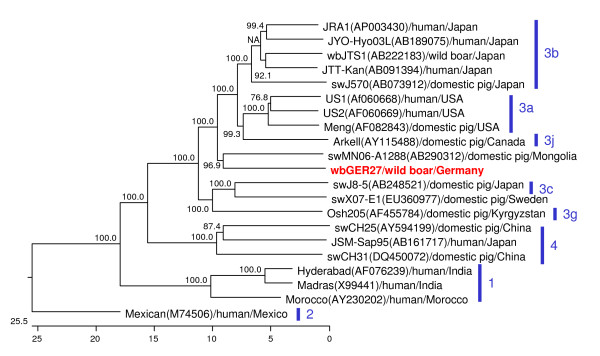
**Comparison of the entire genome sequence of the wild boar isolate wbGER27 with 20 full-length sequences of HEV**. Strain designations, accession numbers, host species and geographical origin of the isolates are indicated. Isolate wbGER27 is shown in red colour. Assigned genotypes are indicated with blue bars. The phylogenetic tree is scaled in nucleotide substitution units.

To get more information about the isolate wbGER27, which was closely related to the human isolate V0706586 and which was nearly identical to the other 8 sequences detected in wild boars from Brandenburg, its whole genome sequence was determined. It consists of 7222 nucleotides (excluding the poly A tail). A BLAST search of the GenBank database using the full-length genome sequence of wbGER27 revealed the highest degree of identity with strain swMN06-A1288, which was originally detected in a pig from Mongolia. This close relationship is also reflected by a phylogenetic tree constructed on the basis of 20 HEV full-length sequences derived from the GenBank database (Figure 4). As no definitive subtype has been assigned to this Mongolian isolate, a grouping of wbGER27 is difficult. However, as it shows only up to 85.3% nucleotide sequence identity to the other isolates and as analysis of the ORF-2 fragment indicated grouping into genotype 3i, this isolate may be considered as the first full-length sequence of genotype 3i. Similar relationships were evident by analysing the deduced amino acid sequences of ORF-1, ORF-2 and ORF-3, with the highest identities of 96.2%, 97.6% and 90.2%, respectively, to those of isolate swMN06-A1288. An analysis of the non-coding regions revealed highly conserved sequences in the 5'-end as well as in the last 23 nucleotides directly adjacent to the poly A tail, but sequence variability in the residual 3' non-coding region.

## Discussion

Our investigations show that HEV is highly prevalent in the German wild boar population with an average detection rate of 14.9% in liver samples. This proportion is higher than that demonstrated in a previous study showing that HEV could be detected in 5.3% of archived German wild boar sera [19]. The differences in detection rates may be explained by the use of different sample material and different storage durations of the samples. A high prevalence of 25% has been also reported for wild boars from Italy, however, only a single population had been investigated in this study [17]. In Japan, several studies reported the detection of HEV or HEV-specific antibodies in the wild boar population leading to the assumption that these animals serve as a reservoir for human HEV infection [9,10,24].

Differences in the determined prevalences may also be caused by the different populations investigated. One of the most obvious finding of our study is the different detection rate in rural vs. urban regions, indicating that a more efficient virus spread among the wild boar population is possible in rural settings. The ecological and/or biological variations between rural vs. urban wild boar populations, which may explain these differences, remain elusive so far. Although with a low number, however, HEV was also detected in urban regions thus indicating that either direct or indirect transmission of HEV from wild boar to humans has to be taken into account in cities also. Notably, the shift from sylvatic to synanthropic occurrence of this game species might lead to a future increase of the infection pressure from HEV on the human population.

We detected a number of different subtypes in the wild boars which clustered due to their geographical origin. This finding argues against short-term epidemics of a certain strain and supports the assumption that several HEV subtypes are endemic in the wild boar population underlining the role of this animal species as a virus reservoir. Clustering of HEV strains according to their geographical distribution has been previously reported for domestic pigs and humans [3,4,11,12]. For domestic pigs in Germany, no data on the prevalence of HEV infection and on the distribution of specific genotypes are available so far. However, in analogy with other European countries [11,22,25,26], a high prevalence of infection with a variety of genotype 3 HEV strains could be expected. Therefore both, domestic pigs and wild boars, have to be considered as reservoirs for HEV in Germany, which may be important for the development of strategies for prevention of HEV infections. In case of wild animals, eradication of the virus infection is more difficult and other groups of the human population have to be considered to be exposed to the virus than in the case of domestic pigs.

Most important, significant homologies were detected between the HEV isolates of wild boars and those derived from autochthonous human cases of hepatitis E, which had been acquired in Germany. Unfortunately, no further information on the distinct geographical origin within Germany or on possible contacts to wild boars was available for these human cases. However, the exceptional high degree of nucleotide sequence homology between the wild boar isolate wbGER27 and the human isolate V0706586 suggests a direct connection between both by direct or indirect (food-borne or by surfaces, environment, or other carrier animals) transmission from wild boar to human. Alternatively, contact of wild boar and human to the same, so far unknown, virus source has to be taken into consideration. The full-length genome sequence of isolate wbGER27 may help to identify further transmission events as it can be compared to any genome fragment generated from a human HEV isolate. Until now, no other HEV full-length sequences derived from wild animals of Europe are available. The generation of more full-length sequences will be necessary due to the detected genetic heterogenicity of the isolates as shown for pigs in Europe [22,26].

## Conclusion

In summary, the results indicate that wild boars may be an important reservoir for HEV in Germany possessing a significant risk for HEV infection of humans. This risk is obvious for hunters, which may be infected during dissection of wild boars. However, consumption of undercooked wild boar meat or contact with faecal contaminations of wild boars may also be taken into consideration. Moreover, HEV was detected with no significant differences in all age groups of wild boars which is in contrast to the situation in domestic pigs, where the age class of 10 to 15 weeks of age is predominantly infected [25,27], thus increasing the risk of virus transmission. The distinct reasons for these differences are not known so far. However, the more intensive contacts between domestic pigs in animal production may explain a more rapid virus spread as compared to the epidemiological situation with rarer contacts between wild boar herds. In Germany, up to 500,000 wild boars are hunted yearly [28], out of these more than 2,000 wild boars in the urban region of Berlin [29]. Further studies on the role of wild boars in the epidemiology of HEV infections are necessary to develop effective measurements for prevention of virus transmission to humans.

## Competing interests

The authors declare that they have no competing interests.

## Authors' contributions

AS carried out the PCR analyses and participated in the sequence alignments. KS and ML participated in the design of the study and collected the samples. BA was included in drafting the manuscript and critical revision. AJ participated in the design of the study and performed the statistical analyses. RJ participated in the design of the study and sequence alignments, and drafted the manuscript.
